# *In silico* identification of isosilybin a targeting squalene epoxidase as an antifungal adjuvant with *in vitro* validation against azole-resistant *Candida* and emerging yeasts

**DOI:** 10.3389/fcimb.2026.1774455

**Published:** 2026-05-12

**Authors:** Akshay Kisan Mundhe, Reena Rajkumari

**Affiliations:** Department of Integrative Biology, School of Bio Sciences and Technology, Vellore Institute of Technology, Vellore, Tamil Nadu, India

**Keywords:** antifungal resistance, azole-resistant yeasts, combination antifungal therapy, ERG1 (squalene epoxidase), in silico pharmacology, isosilybin A, natural antifungal compounds

## Abstract

The rising incidence of antifungal resistance in *Candida* species and emerging non-albicans yeasts has markedly reduced the efficacy of azole antifungal therapy, highlighting the need for resistance modifying strategies. Natural products represent a valuable reservoir of bioactive scaffolds, and computational prioritization facilitates the systematic identification of pharmacologically relevant lead molecules. In this study, we employed an integrated *in silico-in vitro* antimicrobial pharmacology approach to identify Isosilybin A as a potential ERG1-targeting antifungal adjuvant and to assess its biological relevance using Isosilybin A enriched plant extract. A comprehensive virtual screening of 17,967 phytochemicals from the Indian Medicinal Plants, Phytochemistry And Therapeutics (IMPPAT) database was performed against an AlphaFold-predicted structure of squalene epoxidase (ERG1) from *Candida albicans*, followed by drug-likeness and ADMET profiling. Seven shortlisted phytochemicals were subjected to 100 ns molecular dynamics simulations. Among these, Isosilybin A demonstrated persistent interactions with key active-site residues of ERG1 and exhibited stable dynamic behavior throughout the simulation. Favorable binding free energy estimated using Molecular Mechanics/Poisson-Boltzmann Surface Area (MM/PBSA) method positioning it as the foremost mechanistically prioritized candidate. To experimentally validate this computational prioritization, Isosilybin A enriched *Silybum marianum* extract was evaluated against azole-resistant *Candida* species and emerging multidrug-resistant yeast isolates. While the extract alone exhibited no detectable antifungal activity, its combination with fluconazole consistently restored azole susceptibility across all tested isolates. Growth inhibition was observed at a minimum inhibitory concentration of 0.78 mg/mL extract combined with 0.78 μg/mL fluconazole, whereas fluconazole monotherapy remained ineffective. Collectively, these findings establish Isosilybin A as a computationally identified ERG1-interacting antifungal adjuvant, with extract-based *in vitro* validation supporting its role in restoring fluconazole efficacy against resistant *Candida* and emerging yeasts. Further studies employing purified compounds are warranted to conclusively delineate the pharmacological contribution of Isosilybin A.

## 1 Introduction

Invasive and superficial fungal infections continue to pose a significant global health burden, particularly among immunocompromised individuals, with *Candida albicans* recognized as one of the predominant causative agents. Azole antifungals remain the cornerstone of therapy for *Candida* infections; however, their clinical efficacy is increasingly compromised by the rapid emergence of antifungal resistance (Aldardeer et al., 2020; Costa-de-Oliveira and Rodrigues, 2020). The growing prevalence of azole-resistant *Candida* species and emerging non-albicans yeasts has resulted in treatment failures and limited therapeutic options, underscoring an urgent need for resistance-modifying strategies rather than reliance on antifungal monotherapy (Aggarwal et al., 2019; Verma et al., 2021). Allylamine antifungals such as terbinafine exert their antifungal activity by inhibiting squalene epoxidase (ERG1), a key enzyme in the ergosterol biosynthesis pathway essential for fungal membrane integrity and viability (Sagatova, 2021). Clinical reports of terbinafine resistance are increasing, frequently associated with mutations in the ERG1 gene, enhanced efflux activity, and adaptive tolerance mechanisms (Gaurav et al., 2021; Mahmood et al., 2025). Importantly, ERG1 inhibition disrupts ergosterol biosynthesis upstream of azole targets, thereby increasing membrane vulnerability and enhancing azole susceptibility, providing a strong mechanistic basis for combination or adjuvant therapy (Sagatova, 2021; Hu et al., 2023).

Resistance to allylamines and azoles has been reported worldwide, with a particularly alarming rise documented in South Asia, including India (Aggarwal et al., 2019; Verma et al., 2021). Given the historical reliance on terbinafine and azoles for dermatophyte and *Candida* infections, the diminishing effectiveness of these agents highlights the necessity for innovative pharmacological approaches that restore antifungal susceptibility rather than replacing existing drugs (Gaurav et al., 2021). One promising strategy to address antifungal resistance is the identification of adjuvant molecules capable of resensitizing resistant fungal pathogens to existing antifungal agents (Hu et al., 2023; Lou et al., 2024). Rather than developing entirely new antifungals, resistance-modifying adjuvants offer a translationally attractive approach with reduced toxicity and faster clinical applicability (Elgharbawy et al., 2020; Khan et al., 2023). Natural products derived from medicinal plants constitute a rich and chemically diverse source of bioactive scaffolds with known antifungal, anti-inflammatory, and immunomodulatory properties (Elgharbawy et al., 2020; Khan et al., 2023; Singha et al., 2025). Recent research utilizing network pharmacology methods have shown that bioactive herbal substances achieve their therapeutic effects via distinct molecular targets and pathways, thereby offering mechanistic understanding of their pharmacological actions (Hu et al., 2023; Lou et al., 2024). Phytochemicals from traditional medicinal plants have demonstrated the ability to interfere with ergosterol biosynthesis and to act synergistically with azoles, including compounds that target ERG1 or related pathways (Wong-Deyrup et al., 2020). Their structural diversity, relative safety, and multi-target potential make phytochemicals attractive candidates for antifungal adjuvant identification (Ali et al., 2024). Additionally, extracts from various medicinal plants have demonstrated direct antifungal activity against pathogenic fungi, highlighting their potential as alternative or adjunct antifungal agents (Akbar, 2020; Guevara-Lora et al., 2020).

Computational approaches provide powerful tools for the systematic identification and prioritization of candidate compounds from large phytochemical libraries (Almihyawi et al., 2022; Abbas et al., 2025). Molecular docking, pharmacokinetic prediction, molecular dynamics (MD) simulations, and post-simulation analyses enable detailed evaluation of protein-ligand interactions, binding stability, and drug-like properties (Dhanasekaran et al., 2024; Abbas et al., 2025). Importantly, *in silico* screening allows rational prioritization of individual bioactive constituents within complex plant matrices, reducing experimental bias and improving mechanistic interpretability prior to biological validation. Such approaches facilitate comparative assessment of apoproteins, ligand-bound complexes, and reference drug interactions, offering insights into whether alternative molecules can achieve comparable or enhanced stabilization of resistance associated targets such as ERG1 (Almihyawi et al., 2022; Masum et al., 2025).

While experimentally derived crystal structures are the definitive benchmark for structural investigations, such data are currently unavailable for numerous fungal proteins, including *C. albicans’* ERG1. In these instances, predicted protein structures offer a crucial alternative for structure-based research. AlphaFold has demonstrated significant precision in predicting protein structures and has found widespread application in computational drug discovery (Jumper et al., 2021; Borkakoti and Thornton, 2023). Nevertheless, predicted models can present uncertainties, particularly in flexible regions or side-chain orientations within binding pockets. To address these limitations, the AlphaFold model used in this research underwent structural assessment through stereochemical validation methods before docking and MD analyses. Despite these constraints, structures derived from AlphaFold provide a useful basis for identifying potential ligand-protein interactions and prioritizing candidate molecules for subsequent experimental validation. Although purified phytochemicals provide definitive pharmacological attribution, their limited commercial availability and cost can restrict early-stage experimental validation (Wink, 2022). In this context, extract-based assays enriched with computationally prioritized compounds offer a pragmatic approach for initial biological confirmation while preserving translational relevance (Elgharbawy et al., 2020; Khan et al., 2023). Importantly, such validation strategies must be interpreted cautiously, with acknowledgment that observed effects may reflect contributions from multiple co-occurring metabolites (Guevara-Lora et al., 2020).

In this study, we employed an integrated *in silico-in vitro* antimicrobial pharmacology framework to identify ERG1-targeting phytochemical adjuvants from Indian medicinal plants and to evaluate their resistance-modifying potential. Using large-scale virtual screening, ADMET profiling, and MD simulations (Abbas et al., 2025), Isosilybin A was computationally prioritized as a strong ERG1-interacting candidate. This prioritization was subsequently validated *in vitro* using an Isosilybin A enriched *Silybum marianum* extract against azole-resistant *Candida* species and emerging multidrug-resistant yeasts.

## 2 Methodology

### 2.1 Protein preparation

The three-dimensional conformation of the target protein ERG1 was obtained from AlphaFold (AF-Q92206) in PDB format. The model was examined for absent residues, and energy reduction was executed with the AMBER FF99 force field in UCSF Chimera (v1.17.3). Structural quality was evaluated using Per-residue pLDDT profiles and Ramachandran plots to verify backbone conformations and confirm structural integrity (Ramachandran et al., 1963). Refined protein structures were subsequently loaded into AutoDock Vina (v1.1.2) (Dhanasekaran et al., 2024). Polar hydrogens were added, non-polar hydrogens were merged, and Kollman charges were assigned. Finalized structures were saved in PDBQT format for the virtual screening of phytochemicals from the IMPPAT library. The AlphaFold-predicted ERG1 structure was selected due to the absence of an experimentally resolved structure and was validated through Ramachandran plot analysis to ensure acceptable stereochemical quality prior to docking.

### 2.2 Ligand preparation

Phytochemicals were obtained from the IMPPAT collection (Vivek-Ananth et al., 2023). A total of 17,967 substances with accessible three-dimensional structures were retrieved from the PubChem database (Kim et al., 2023). The compounds underwent pre-processing with OpenBabel, which involved hydrogen addition and energy reduction with the MMFF94 force field through the conjugate gradient technique (Halgren, 1996). Optimized structures were saved in MOL2 format (O’Boyle et al., 2011) and later transformed into PDBQT format with the mol2s_to_pdbqts.sh script (Li and Shah, 2017).

### 2.3 Prediction of active sites

Potential binding sites of ERG1 were predicted utilizing the CASTp web server (http://sts.bioe.uic.edu/castp/calculation.html), which finds topological pockets and cavities (Binkowski et al., 2003). Literature evidence was further employed to corroborate the functional importance of the anticipated residues implicated in ligand binding.

### 2.4 Optimization of docking configuration

Docking simulations were conducted using AutoDock Vina (version 1.1.2). The docking grid was established with coordinates X = –5.278, Y = –0.431, Z = –1.159, and size of 30 × 30 × 30 Å. Preliminary simulations were conducted with an energy range of 3, an exhaustiveness level of 8, and a maximum of 9 docking modes. For the leading 500 phytochemicals, the exhaustiveness was elevated to 50 and the docking ways to 20 to enhance sampling precision (**Supplementary Table 1** in **Supplementary File 1**). AutoDock Vina produced up to 20 conformations for each ligand, sorted by binding affinity, with the conformation exhibiting the lowest binding energy being most advantageous. Docked complexes were viewed and analyzed utilizing UCSF Chimera (v1.17.3) and Discovery Studio 2021. Higher exhaustiveness and increased docking modes were applied to the top-ranked phytochemicals to enhance conformational sampling accuracy while maintaining computational efficiency during large-scale screening.

### 2.5 Prediction of drug-likeness

The drug-likeness, pharmacokinetics, and physicochemical features of the top 49 ligands were assessed utilizing SwissADME (http://www.swissadme.ch). Essential instruments like the BOILED-Egg model, Bioavailability Radar, and iLOGP were utilized for profiling (Daina et al., 2017). Furthermore, ADMETlab 2.0 (https://admetlab3.scbdd.com/server/evaluationCal) was utilized to forecast toxicity and bioavailability metrics. Ligands were shortlisted sequentially based on docking score, active-site interactions, Lipinski compliance, and predicted ADMET properties, resulting in seven candidates selected for MD simulations (Daina et al., 2017; Dhanasekaran et al., 2024).

### 2.6 Molecular dynamics simulations

MD simulations were performed for ERG1 in its apo form and in complex with the seven ligands exhibiting the lowest binding energy and stable hydrogen bonding, as well as the reference medication terbinafine. Simulations were conducted with GROMACS (v2022.5) with the CHARMM force field for duration of 100 ns (Schüttelkopf and Van Aalten, 2004; Dhanasekaran et al., 2024). Energy reduction was accomplished by the steepest descent method, involving 1264 steps. The system was solubilized in a cubic box containing TIP3P water molecules, with the addition of 0.15 M NaCl to achieve system neutrality. Equilibration was conducted under NVT and NPT ensembles for 1000 ps each, succeeded by a 100 ns production phase (Prakash et al., 2020; Dhanasekaran et al., 2024).

Trajectory investigations encompassed root-mean-square deviation (RMSD), root-mean-square fluctuation (RMSF), radius of gyration (Rg), solvent-accessible surface area (SASA), and intermolecular hydrogen bonding (Hb), utilizing standard GROMACS utilities (gmx rmsd, gmx rmsf, gmx gyrate, gmx sasa, gmx hbond). The output data were viewed using XMGrace (version 5.1.25) (Maurya and Mishra, 2022; Benjamin et al., 2023; Dhanasekaran et al., 2024). All quantitative analyses were performed on the equilibrated production phase (20–100 ns) to exclude initial system relaxation effects.

Additionally, Principal Component Analysis (PCA), also known as essential dynamics, was performed to investigate the dominant collective motions within the protein–ligand complexes. The covariance matrices of Cα atomic positional fluctuations were generated using gmx covar, and diagonalized with gmx anaeig to obtain eigenvectors and eigenvalues. Trajectories were then projected onto the principal components (PC1–PC3) to evaluate conformational sampling and characterize large-scale motion patterns during the simulation. Binding free energy calculations were performed using the Molecular Mechanics/Poisson Boltzmann Surface Area (MM/PBSA) method to estimate the stability of ligand-protein complexes based on MD trajectories.

### 2.7 Extraction and verification of phytochemicals from *Silybum marianum*

**Figure 1:**
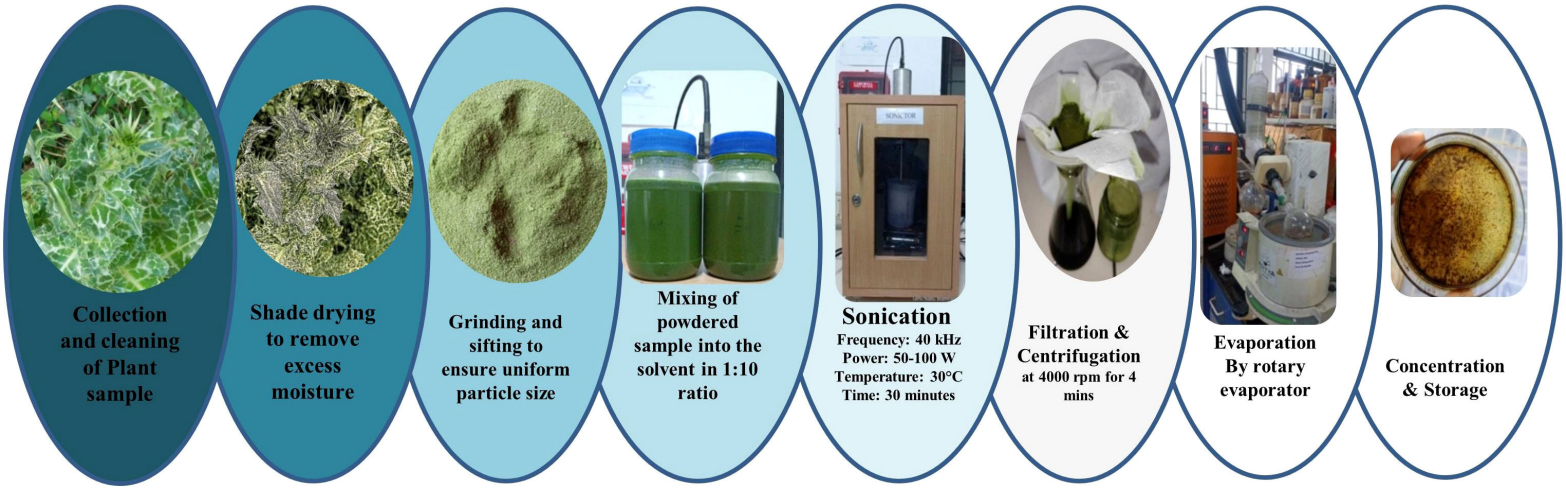
Schematic representation of the extraction and concentration procedure for milk thistle plant extract involving collection, drying, grinding, solvent mixing, sonication, centrifugation, rotary evaporation, and storage.

Isosilybin A was chosen for experimental validation due to its robust and consistent interactions seen during MD simulations among the phytochemicals. Seeds of *S. marianum* were purified, desiccated, and finely milled. Solvent extraction was conducted individually utilizing 75% ethanol and 75% methanol at a 1:8 (w/v) ratio, accompanied by ultra-sonication (40 kHz, 30 minutes) to enhance phytochemical liberation (Zhang et al., 2024; Tan et al., 2025). The extracts were filtered and centrifuged at 4000 rpm for 4 minutes, after which the resultant supernatants were concentrated using a rotary evaporator (**Figure 1**). Isosilybin A and other phytochemicals were verified using liquid chromatography-high-resolution mass spectrometry (LC-HRMS) based on accurate mass and fragmentation pattern. However, absolute quantification of Isosilybin A was not performed in the present study due to the absence of a reference standard.

### 2.8 *In vitro* evaluation of antifungal activity

The preliminary antifungal efficacy of the *S. marianum* extract was assessed utilizing the agar well diffusion method. Mueller-Hinton agar (MHA) plates, augmented with yeast extract and chloramphenicol, were inoculated with standardized yeast suspensions of *C. albicans* (ATCC 10231) as the reference strain, in addition to environmental isolates of *C. albicans*, non-albicans *Candida* species, and other relevant yeast pathogens, as detailed in **Table 1**. The environmental isolates were *C. tropicalis* (VITAKREE1), *Naganishia randhawae* (VITAKREE2), *Trichosporon asahii* (VITAKREE3), *Diutina rugosa* [syn. *C. rugosa*] (VITAKREE7), *C. albicans* (VITAKREE8), *C. tropicalis* (VITAKREE19), and *Pichia kudriavzevii* (VITAKREE20 and VITAKREE23) (https://submit.ncbi.nlm.nih.gov/).

**Table 1:** List of fungal isolates tested against *S. marianum* extract for *in vitro* antifungal validation, including standard and environmental strains with corresponding GenBank submission codes.

Isolate type	Isolate code	Organism	strain code submitted in NCBI genbank database
Standard isolate	C	*C. albicans-*ATCC10231	
Environmental isolates	1	*C. tropicalis*	VITAKREE1
	2	*Naganishia randhawae*	VITAKREE2
	3	*Trichosporon asahii*	VITAKREE3
	7	*Diutina rugosa/C. rugosa*	VITAKREE7
	8	*C. albicans*	VITAKREE8
	19	*C. tropicalis*	VITAKREE19
	20	*Pichia kudriavzevii*	VITAKREE20
	23	*Pichia kudriavzevii*	VITAKREE23

A total of nine fungal isolates were evaluated, including one standard reference strain (*C. albicans* ATCC 10231) and nine environmental isolates obtained from bird excreta, sewage water, and soil samples.

A concentrated stock solution of the crude *S. marianum* extract (1000 mg/mL) was formulated in 25% dimethyl sulfoxide (DMSO). The stock was diluted with sterile distilled water to achieve an initial working concentration of 50 mg/mL, equating to roughly 1.25% DMSO. Subsequently, a series of two-fold serial dilutions were conducted to achieve seven final working concentrations (50, 25, 12.5, 6.25, 3.125, 1.56, and 0.78 mg/mL) for minimum inhibitory concentration (MIC), each sustaining a final DMSO content of 1.25%. For preliminary qualitative antifungal assay against each test organism, four wells were filled with first four concentrations of extract, while a fifth center well functioned as the solvent control containing 1.25% DMSO only.

Fluconazole doses utilized for comparative and combinatorial experiments were individually produced in sterile distilled water. The initial working concentration was 50 μg/mL, which was serially diluted in two-fold steps to yield seven concentrations (from 50 to 0.78 μg/mL) for MIC assessment. The same first four concentrations were used for preliminary agar well diffusion experiments in drug alone as well as in conjunction with the *S. marianum* extract.

All isolates were previously verified to demonstrate resistance to standard antifungal agents amphotericin B, caspofungin, fluconazole, and flucytosine utilizing commercial antifungal susceptibility kits. The antifungal effectiveness of the *S. marianum* extract was assessed both alone and in conjunction with fluconazole to examine potential synergistic effects. The fluconazole-extract combination was evaluated against the documented synergistic interaction between terbinafine and fluconazole, which is known to increase fluconazole sensitivity in resistant *Candida* strains (Khodavandi et al., 2014). After initial screening using agar well diffusion method, MIC experiments were conducted to quantitatively assess the antifungal efficacy of fluconazole both individually and in conjunction with the Isosilybin A enriched *S. marianum* extract. Two-fold serial dilutions of the extract were performed, extending to the seventh dilution (0.78 mg/mL). Each well of a sterile 96-well microtiter plate was filled with 100 μL of the antifungal solution (fluconazole alone in Set A and combination of both drug plus Isosilybin A rich extract in Set B), 100 μL of double-strength Mueller-Hinton broth enriched with yeast extract and chloramphenicol, and 10 μL of standardized yeast inoculum (0.5 McFarland standard; about 1 × 10³ CFU/mL). Plates were incubated at 37 °C for 24 hours. Although stock solutions were prepared in 25% DMSO, serial dilution ensured that the final DMSO concentration in the test wells did not exceed 1.25%, a level that showed no inhibitory effect in solvent control wells. During the broth microdilution MIC assay, the final DMSO concentration was further reduced by 50%, as each well contained an equal volume of double-strength Mueller-Hinton broth, resulting in two-fold dilution of the test solutions.

Following incubation, 10 μL of resazurin solution (0.1 mg/mL) was introduced to each well, resulting in a final concentration of 0.005 mg/mL. The plates were subsequently incubated for 2 hours at 37 °C, and colorimetric changes were visually evaluated to ascertain cell viability. Wells exhibiting no color change (blue) were deemed suggestive of total growth inhibition, whereas pink wells signified viable growth.

All isolates exhibited resistance to conventional antifungal agents amphotericin B, caspofungin, fluconazole, and flucytosine based on MIC assays performed using commercial antifungal susceptibility testing kits. The observed enhancement of fluconazole activity in the presence of the extract was interpreted as a synergistic interaction based on consistent MIC reduction across all isolates. As the *in vitro* validation was performed using an Isosilybin A enriched extract rather than the purified compound, the contribution of additional metabolites cannot be entirely excluded. All the antifungal experiments including qualitative and quantitative were triplicated and the average values were used for analysis.

## 3 Result

### 3.1 Virtual screening and docking

**Figure 2:**
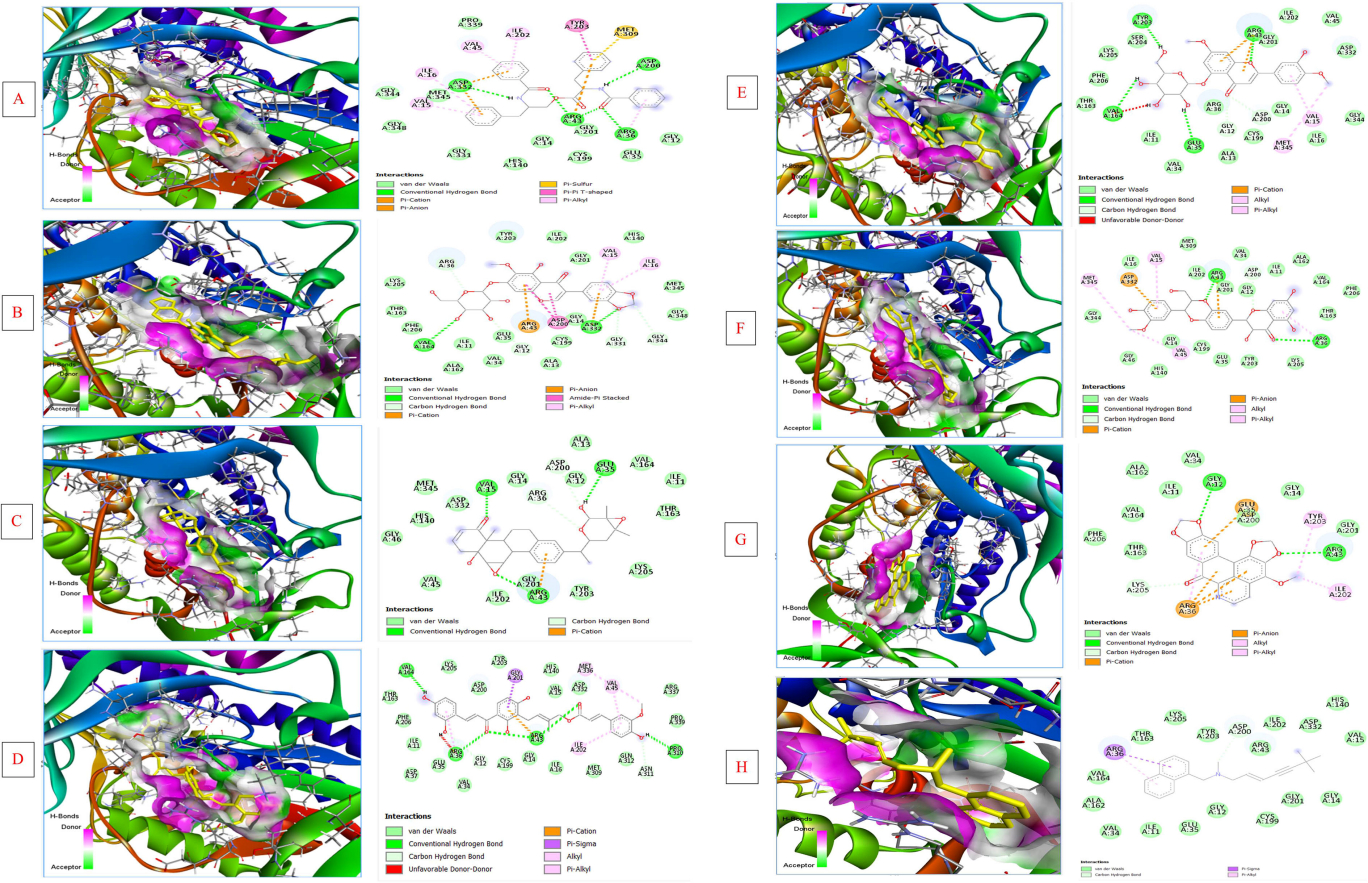
Docked poses and 2D interaction maps of top phytochemical hits with ERG1. **(A)** Asperphenamate, **(B)** Dalspinin-7-O-β-D-galactopyranoside, **(C)** SCHEMBL17241083, **(D)** Isogemichalcone **(C)** Docked poses and 2D interaction maps of top phytochemical hits with ERG1. **(E)** Pilloin 5-glucoside, **(F)** Isosilybin A, **(G)** Cassamedine, **(H)** Terbinafine. Protein shown in cartoon, ligand as sticks; hydrogen bonds shown as dashed lines and hydrophobic contacts highlighted. Key interacting residues predicted by docking are labeled. (See **Supplementary Table 4** in **Supplementary File 1** for interacting residues).

From an initial screen of 17,967 phytochemicals from the IMPPAT library, the top-scoring compounds against the AlphaFold model of *C. albicans* ERG1 were selected for further analysis (**Table 2**). Docking with AutoDock Vina yielded several ligands with predicted binding energies ≤ −10 kcal/mol (Asperphenamate, Dalspinin-7-O-β-D-galactopyranoside, SCHEMBL17241083, Isogemichalcone C, Pilloin 5-glucoside, Isosilybin A and Cassamedine). Inspection of docking poses and interaction maps (**Figure 2**) indicates that the high-affinity phytochemicals occupy the predicted catalytic/pocket region of ERG1 and form a combination of polar contacts (Arg36, Arg43, Asp200, Asp332, Val164, and adjacent residues) and extensive hydrophobic packing with nearby residues. Terbinafine, included as reference, showed a higher docking score than the top phytochemicals but engages several pocket residues consistent with its known mode of action. However, the docking scores offer only a rough approximation of ligand-protein affinity, and variations of a few kcal/mol might not directly correlate with significant biological activity. Consequently, the docking outcomes were utilized primarily as an initial screening procedure to rank candidate molecules for subsequent MD simulations and experimental assessment.

**Table 2:** Molecular weight, IMPPAT ID, docking score, and source plants of the leading phytochemicals.

Phytochemical name	ml wt	IMPPAT ID	Docking score (kcal/mol)	Source plant
Asperphenamate	506.6	IMPHY005141	-10.7	*Medicago lupulina*
Dalspinin-7-O-beta-D-galactopyranoside	490.42	IMPHY012841	-10.5	*Dalbergia spinosa*
SCHEMBL17241083	466.57	IMPHY010989	-10.4	Not available
Isogemichalcone C	532.55	IMPHY002761	-10.3	*Broussonetia papyrifera*
Pilloin 5-glucoside	476.43	IMPHY004337	-10.1	*Prunus persica*
Isosilybin A	482.44	IMPHY011702	-10.1	*Silybum marianum*
Cassamedine	349.3	IMPHY002118	-10.1	*Cassytha filiformis*
Control Drug	ml wt	IMPPAT ID	Docking Score	Source
Terbinafine	291.4	not applicable	-8.77	Synthetic compound

All top seven phytocompounds displayed persistent and diversified binding patterns in the ERG1 active site, promoted by van der Waals, hydrophobic, electrostatic, and hydrogen bond interactions (**Figures 2A, B**) (**Supplementary Table 2** in **Supplementary File 1**). Isogemichalcone C and Cassamedine have the strongest van der Waals and polar contacts, showing strong binding pocket complementarity. ILE11, GLY12, GLY14, GLU35, ARG36, ARG43, CYS199, GLY201, ILE202, TYR203, LYS205, ASP200, and ASP332 consistently interacted across many complexes, indicating their role in ligand stabilization. In compounds like Cassamedine, Isogemichalcone C, and Isosilybin A, carbon-hydrogen bonds increased complex stability. ARG36, ARG43, ASP200, and ASP332 largely had conventional hydrogen bonding. Isogemichalcone C has a balanced polar–nonpolar binding profile, forming hydrogen bonds with ARG36, ARG43, and VAL164 and van der Waals interactions with hydrophobic residues. Isosilybin A and SCHEMBL17241083 showed dual hydrogen bond properties, indicating high-affinity ligand potential. Terbinafine, the benchmark antifungal, exhibited fewer polar contacts and one carbon-hydrogen bond with ASP200, indicating a weaker connection. The interaction landscape shows that Isogemichalcone C, Cassamedine, and Isosilybin A exhibited superior predicted binding affinity and favorable interaction profiles with ERG1 compared to the reference drug terbinafine. Natural products sourced from medicinal plants have been extensively studied for their varied biological activities, encompassing both antimicrobial and anticancer properties. As an illustration, polysaccharides extracted from plants have exhibited notable biological activity and advantageous physicochemical characteristics *in vitro* (Xiao et al., 2025). Consequently, these observations bolster the investigation of plant-derived molecules as prospective therapeutic agents.

### 3.2 Drug-likeness and ADMET profiling

The study began with 17,967 phytochemicals from the IMPPAT database. Based on molecular docking against ERG1, the top 500 compounds were retained, as binding energies remained favorable within this range. From these, 49 molecules were further shortlisted through visual inspection in Discovery Studio, ensuring that candidate ligands formed hydrogen-bonding interactions with active-site residues of ERG1.

To further refine this set, drug-likeness filters were applied prior to MD simulations. Specifically, Lipinski’s Rule of Five (Ro5) was used as the primary criterion for advancing compounds into dynamic simulations, as compliance with Ro5 is a fundamental predictor of oral drug-likeness. This step reduced the set to seven phytochemicals, which were then subjected to comprehensive ADMET profiling and MD analysis.

SwissADME and ADMETLab predictions (see **Table 3** in **Supplementary File 1**) revealed a spectrum of pharmacokinetic properties. Asperphenamate satisfied Ro5 but failed Ghose, Veber, Pfizer and GSK filters, coupled with poor solubility and a very low bioavailability score (0.17). Dalspinin-7-O-β-D-galactopyranoside also passed Ro5, with good solubility predictions, but failed Ghose and Veber filters and was predicted to have low GI absorption. SCHEMBL17241083 passed Lipinski, Ghose and Veber filters, exhibited moderate solubility and a bioavailability score of 0.55, although it failed Pfizer and GSK filters. Isogemichalcone C passed Ro5 but failed the other major filters, with poor-to-moderate solubility and low GI absorption. Pilloin-5-glucoside passed Lipinski and Ghose filters and was soluble in both ESOL and Silicos-IT models, though it failed the Veber filter and had low GI absorption. Isosilybin A satisfied Ro5, displayed moderate solubility, and achieved an acceptable bioavailability score (0.55), despite failing Ghose and Veber filters. Cassamedine was the strongest ADMET candidate, passing all five drug-likeness filters (Lipinski, Ghose, Veber, Pfizer, GSK) with moderate solubility, high GI absorption, predicted BBB permeability, and a bioavailability score of 0.55.

Consequently, although Lipinski compliance was the primary selection criterion for the next MD simulations, the comprehensive ADMET profile offered vital supplementary insights for candidate prioritizing. Compounds like Cassamedine, which successfully met all five drug-likeness criteria (Lipinski, Ghose, Veber, Pfizer, and GSK) and exhibited advantageous pharmacokinetic properties, had been expected to be attractive for subsequent dynamic assessment. Likewise, phytochemicals such as Isosilybin A and Pilloin-5-glucoside, which exhibited satisfactory pharmacokinetic characteristics and bioavailability despite restricted filter compliance, were also selected. This comprehensive methodology, encompassing large-scale docking, Ro5-based screening, and ADMET assessment, facilitated the judicious selection of natural product candidates for in-depth molecular dynamics research targeting *C. albicans* ERG1. Computational ADMET predictions provide valuable insights into the *in vivo* behavior of prospective molecules; however, formulation and delivery strategies frequently prove essential for enhancing the bioavailability and therapeutic efficacy of natural compounds. For instance, micelle-based drug delivery systems have been shown to improve the pharmacokinetic profiles and biological effectiveness of plant-derived compounds, including puerarin (Li W. et al., 2018). Moreover, targeted micellar formulations, modified with triphenylphosphonium cations, have exhibited improved cellular targeting and biological protection (Li et al., 2019). Recent developments in drug conjugation techniques, which facilitate extended drug delivery and improved pharmacological stability, have also been documented for targeted therapeutic agents (Zheng et al., 2025). These approaches underscore potential methods for augmenting the pharmacological effectiveness of promising phytochemicals identified through computational screening.

### 3.3 Molecular dynamics simulation

#### 3.3.1 Molecular dynamics: global stability (RMSD, Rg, SASA)

**Figure 3:**
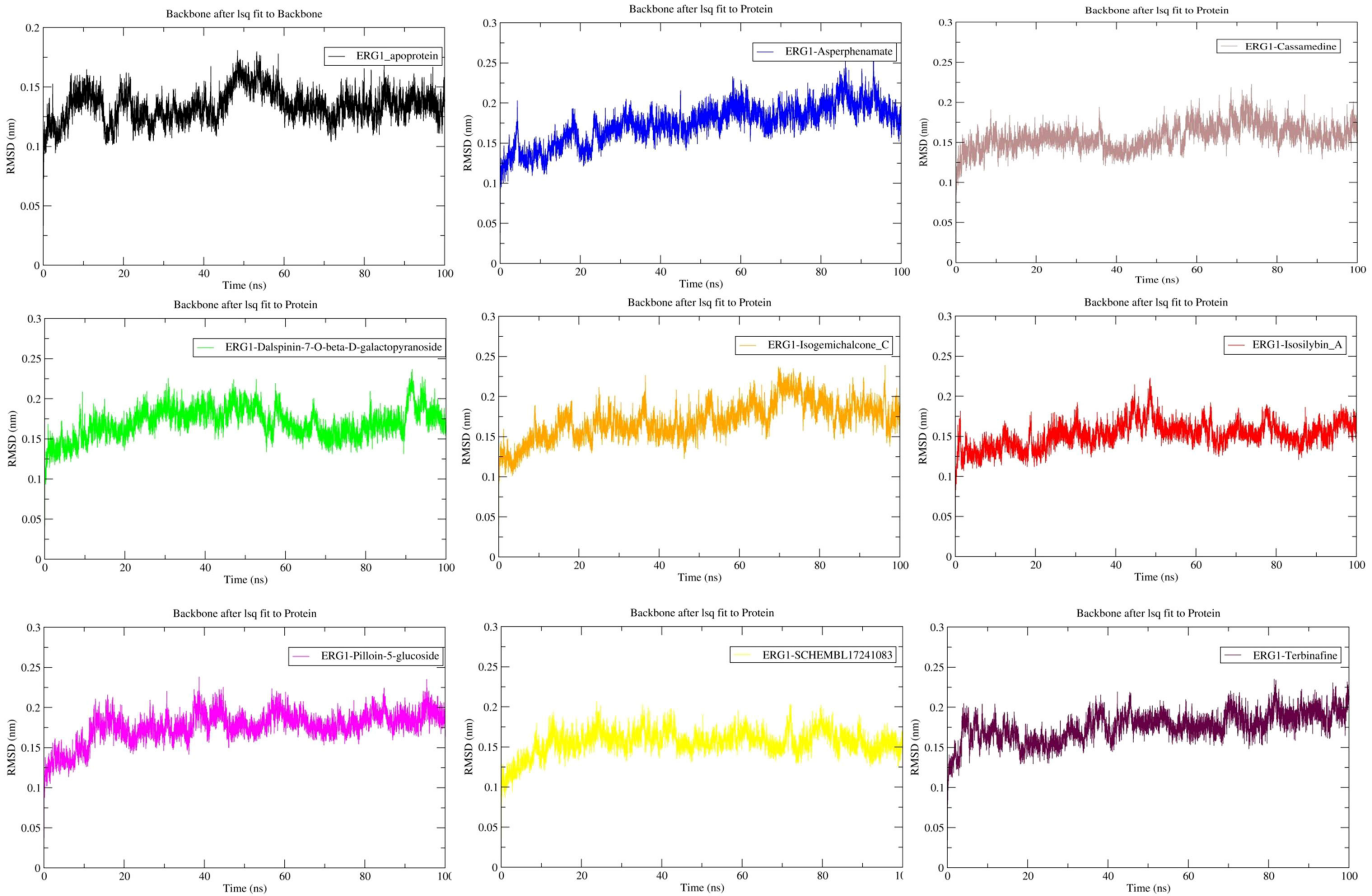
Backbone RMSD time-series for apo ERG1 and ligand-bound complexes. RMSD (nm) was calculated after least-squares fitting to the initial protein backbone structure. Shaded regions indicate the equilibration period (0–20 ns); reported analyses focus on the production window (20–100 ns). Values reported are mean ± SD over 20–100 ns: For apoprotein = 0.135 ± 0.012 nm; Asperphenamate-protein = 0.182 ± 0.018 nm; Cassamedine-protein = 0.160 ± 0.015 nm; Dalspinin-7-O-β-D-galactopyranoside-protein = 0.175 ± 0.016 nm; Isogemichalcone C-protein = 0.177 ± 0.019 nm; Isosilybin A-protein = 0.157 ± 0.014 nm; Pilloin-5-glucoside-protein = 0.182 ± 0.013 nm; SCHEMBL17241083-protein = 0.160 ± 0.012 nm; Terbinafine-protein = 0.180 ± 0.016 nm. Equilibration period (0–20 ns) shaded (**Table 3**).

Backbone RMSD analyses (Cα/backbone) were computed over the production window (20–100 ns) (**Figure 3**). The apo ERG1 structure remained highly stable with a mean RMSD of 0.135 ± 0.012 nm. Most ligand-bound systems showed slightly higher mean RMSD values: Asperphenamate-ERG1 (0.182 ± 0.018 nm), Pilloin-5-glucoside-ERG1 (0.182 ± 0.013 nm), Terbinafine-ERG1 (0.180 ± 0.016 nm) and Isogemichalcone C-ERG1 (0.177 ± 0.019 nm). Isosilybin A-ERG1 and SCHEMBL17241083-ERG1 showed intermediate stability (0.157 ± 0.014 nm and 0.160 ± 0.012 nm, respectively), while Cassamedine-ERG1 and Dalspinin-7-O-β-D-galactopyranoside-ERG1 complexes were near 0.16–0.175 nm. Overall, all systems reached a stable plateau after ~10–20 ns and retained the global fold for the remainder of the simulations; a few compound bound complexes (notably Asperphenamate-ERG1 and Pilloin-5-glucoside-ERG1) showed modestly higher RMSD and slightly larger fluctuations, suggesting small global rearrangements but no loss of overall fold (**Table 3**).

**Table 3:** Average global stability metrics (backbone RMSD, Rg, SASA) mean ± SD (20–100 ns).

System	Backbone RMSD (Cα) (nm)	Radius of gyration Rg (Cα) (nm)	SASA (nm²)
Apo (ERG1)	0.135 ± 0.012	2.339 ± 0.006	217.72 ± 3.06
Asperphenamate	0.182 ± 0.018	2.366 ± 0.008	224.81 ± 3.08
Cassamedine	0.160 ± 0.015	2.360 ± 0.009	225.42 ± 2.70
Dalspinin-7-O-β-D-galactopyranoside	0.175 ± 0.016	2.356 ± 0.008	222.07 ± 2.88
Isogemichalcone C	0.177 ± 0.019	2.361 ± 0.007	225.18 ± 3.09
Isosilybin A	0.157 ± 0.014	2.343 ± 0.006	222.16 ± 2.63
Pilloin-5-glucoside	0.182 ± 0.013	2.353 ± 0.009	225.50 ± 3.00
SCHEMBL17241083	0.160 ± 0.012	2.350 ± 0.008	224.06 ± 2.68

Values are mean ± SD calculated over the production window (20–100 ns). Backbone RMSD is for Cα/backbone (nm); Rg is radius of gyration computed on Cα (nm); SASA is solvent-accessible surface area (nm²). Frame count per trajectory ≈ 8000 (sampling interval as in original analysis). Equilibration window (0–20 ns) excluded.

**Figure 4:**
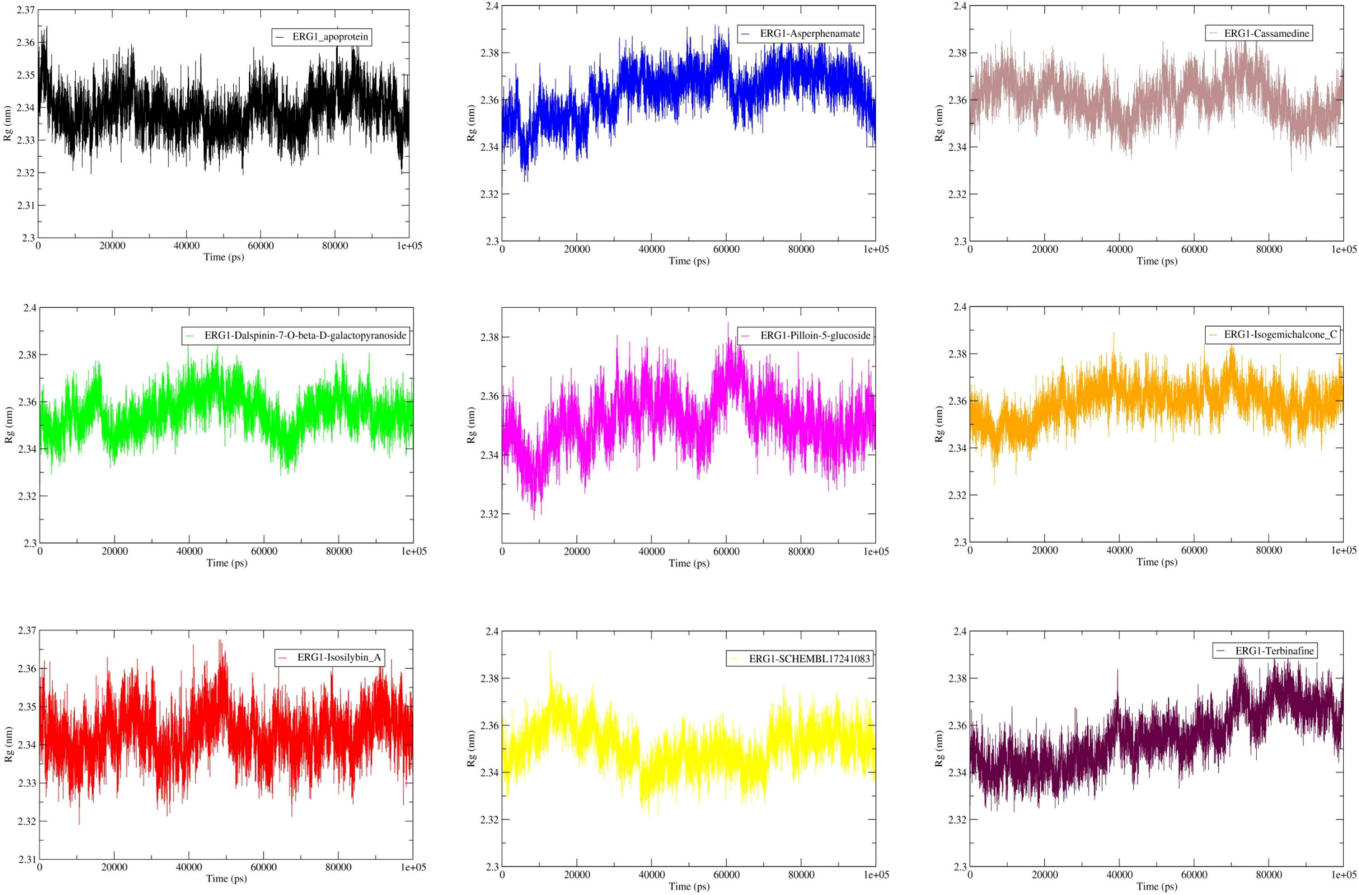
Radius of gyration (Rg) for apo and ligand-bound ERG1 over 100 ns. Rg (nm) computed from backbone atoms shows the overall compactness of the protein remains stable across systems. Note: Values reported are mean ± SD over 20–100 ns: For apo protein = 2.339 ± 0.006 nm; Asperphenamate-protein = 2.366 ± 0.008 nm; Cassamedine-protein = 2.360 ± 0.009 nm; Dalspinin-7-O-β-D-galactopyranoside-protein = 2.356 ± 0.008 nm; Isogemichalcone C-protein = 2.361 ± 0.007 nm; Isosilybin A-protein = 2.343 ± 0.006 nm; Pilloin-5-glucoside-protein = 2.353 ± 0.009 nm; SCHEMBL17241083-protein = 2.350 ± 0.008 nm; Terbinafine-protein = 2.358 ± 0.011 nm. Equilibration (0–20 ns) shaded (**Table 3**).

Radius of gyration (Cα) remained essentially constant across all systems over the production window (20–100 ns) (**Figure 4**). Mean Rg values ranged narrowly from ~2.34 nm (apo) to ~2.37 nm (Asperphenamate), with small standard deviations (≤0.012 nm), indicating no substantial compaction or expansion of ERG1 upon ligand binding. Isosilybin A shows Rg (2.343 ± 0.006 nm) very close to apo, consistent with little global rearrangement, whereas Asperphenamate displays the largest mean Rg (2.366 ± 0.008 nm), matching the modestly higher backbone RMSD previously reported for that complex (**Table 3**).

**Figure 5:**
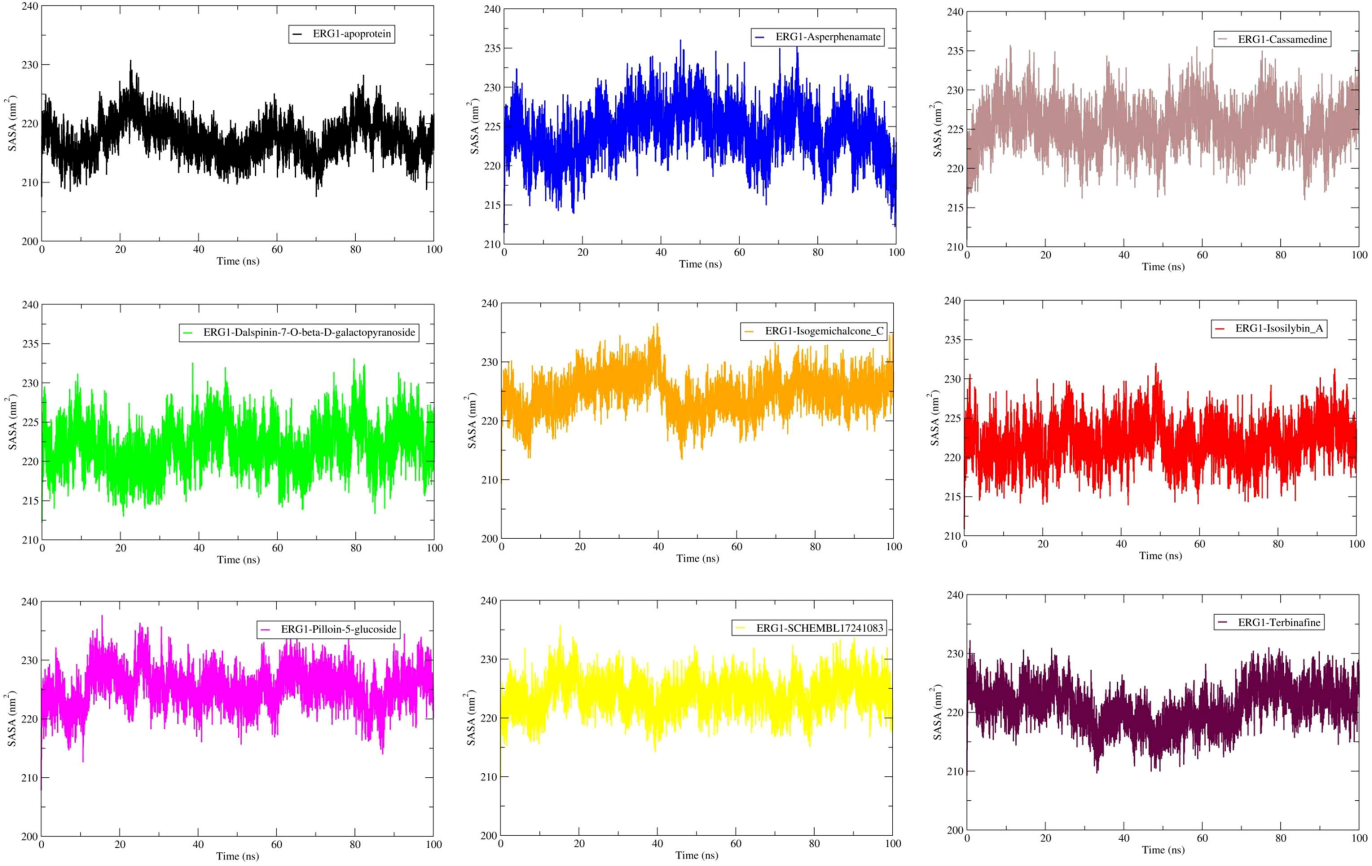
Solvent-accessible surface area (SASA) for apo and ligand complexes (100 ns). Mean ± SD values (20–100 ns): For apoprotein = 217.72 ± 3.06 nm²; Asperphenamate-protein = 224.81 ± 3.08 nm²; Cassamedine-protein = 225.42 ± 2.70 nm²; Dalspinin-7-O-β-D-galactopyranoside-protein = 222.07 ± 2.88 nm²; Isogemichalcone C-protein = 225.18 ± 3.09 nm²; Isosilybin A-protein = 222.16 ± 2.63 nm²; Pilloin-5-glucoside-protein = 225.50 ± 3.00 nm²; SCHEMBL17241083-protein = 224.06 ± 2.68 (**Table 3**).

SASA time-series were analyzed over the production window (20–100 ns) (**Figure 5**). The apo ERG1 shows a mean SASA of 217.72 ± 3.06 nm², while ligand-bound systems span 222.07–225.50 nm². The largest mean solvent exposure was observed for Pilloin-5-glucoside-ERG1 (225.50 ± 3.00 nm²) and Cassamedine-ERG1 (225.42 ± 2.70 nm²) bound complexes, whereas the values for Isosilybin A-ERG1 (222.16 ± 2.63 nm²) and Dalspinin-7-O-β-D-galactopyranoside-ERG1 (222.07 ± 2.88 nm²) were closer to apoprotein. Overall, SASA values are tightly clustered (SD ≤ ~3.5 nm²), indicating no major ligand-induced changes in global solvent exposure and supporting preservation of the protein fold across the complexes (**Table 3**).

#### 3.3.2 Molecular dynamics: local flexibility (RMSF)

**Figure 6:**
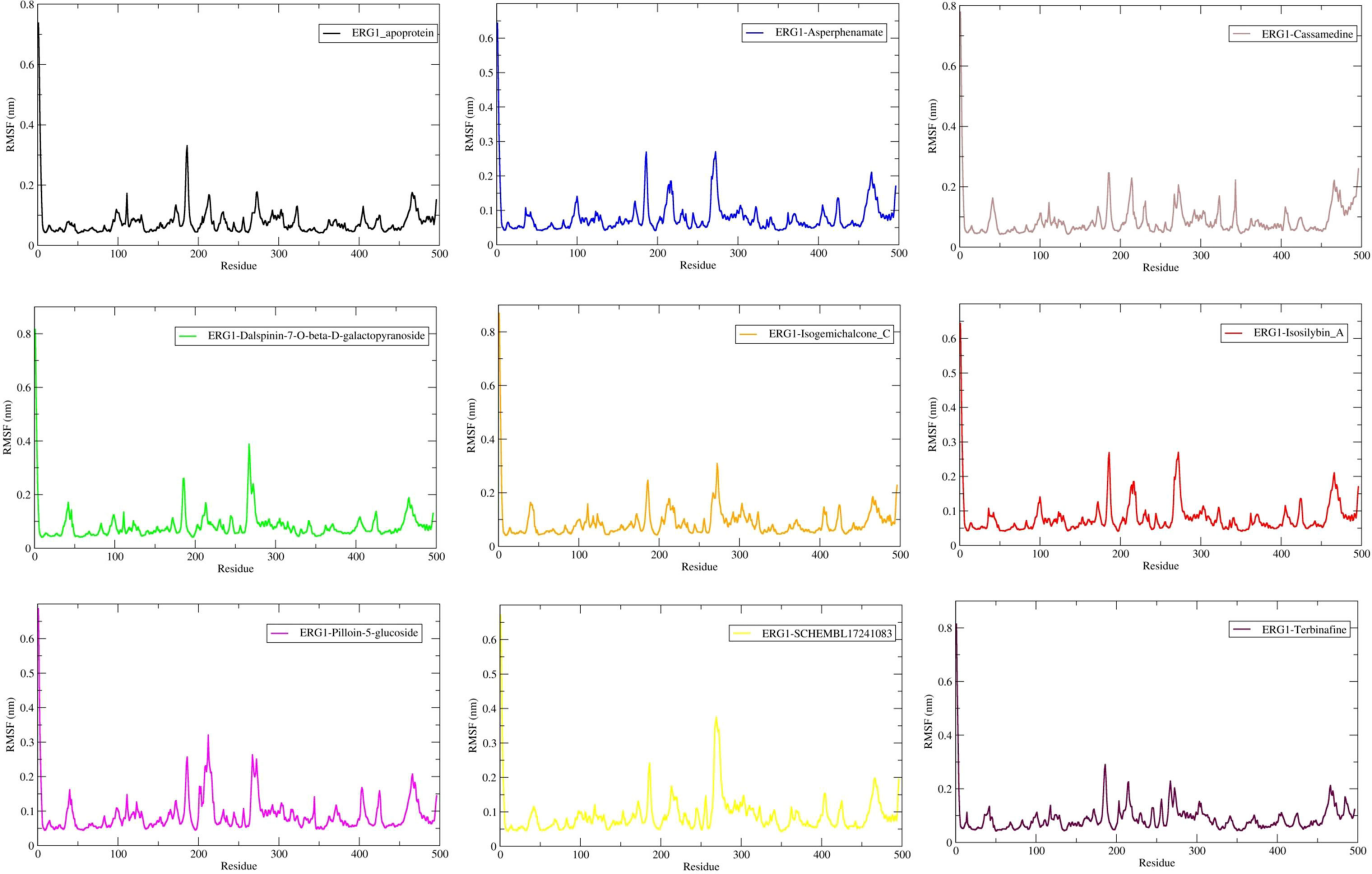
Per-residue root-mean-square fluctuation (RMSF) for apo ERG1 and ligand complexes over 100 ns. Values are RMSF on Cα atoms (nm). Mean RMSF across all residues (20–100 ns) are: For apoprotein = 0.0769 ± 0.0082 nm; Asperphenamate-protein = 0.0794 ± 0.0091 nm; Cassamedine-protein = 0.0867 ± 0.0114 nm; Dalspinin-7-O-β-D-galactopyranoside-protein = 0.0830 ± 0.0113 nm; Isogemichalcone C-protein = 0.0869 ± 0.0121 nm; Isosilybin A-protein = 0.0794 ± 0.0091 nm; Pilloin-5-glucoside-protein = 0.0888 ± 0.0131 nm; SCHEMBL17241083-protein = 0.0897 ± 0.0114 nm; Terbinafine-protein = 0.0890 ± 0.0112 nm. Pocket-region RMSF (Arg36, Arg43, Val164, Asp200, and Asp332) is shown in the main text (**Table 4**).

Per-residue backbone RMSF analysis (20–100 ns) indicates that loop regions and terminal residues remain the most flexible segments in ERG1 across apoprotein and ligand-bound forms (**Figure 6**). Mean RMSF across all residues ranged from 0.0769 ± 0.0082 nm (apo) to 0.0897 ± 0.0114 nm (SCHEMBL17241083-ERG1). RMSF averaged over pocket residues (Arg36, Arg43, Val164, Asp200, Asp332) remained low and broadly similar across systems (e.g., apoprotein = 0.0624 ± 0.0102 nm; Isosilybin A-ERG1 = 0.0724 ± 0.0227 nm), indicating the binding site is not highly mobile in any complex and in some ligand complexes shows modest local stabilization (**Table 4**).

**Table 4:** Average backbone RMSF and pocket-region RMSF for ERG1 and ligand complexes (20–100 ns).

System	Mean RMSF (nm) ± SD	Pocket RMSF mean (nm) ± SD
Apo (ERG1)	0.07686 ± 0.00820	0.06238 ± 0.01024
Asperphenamate	0.07938 ± 0.00905	0.07240 ± 0.02272
Cassamedine	0.08671 ± 0.01141	0.08154 ± 0.02518
Dalspinin-7-O-β-D-galactopyranoside	0.08300 ± 0.01125	0.08426 ± 0.03225
Isogemichalcone C	0.08685 ± 0.01209	0.08266 ± 0.03222
Isosilybin A	0.07938 ± 0.00905	0.07240 ± 0.02272
Pilloin-5-glucoside	0.08875 ± 0.01308	0.09106 ± 0.00734
SCHEMBL17241083	0.08973 ± 0.01143	0.07766 ± 0.01834
Terbinafine	0.08895 ± 0.01119	0.08010 ± 0.01822

#### 3.3.3 Intermolecular hydrogen bonding and binding interactions

**Figure 7:**
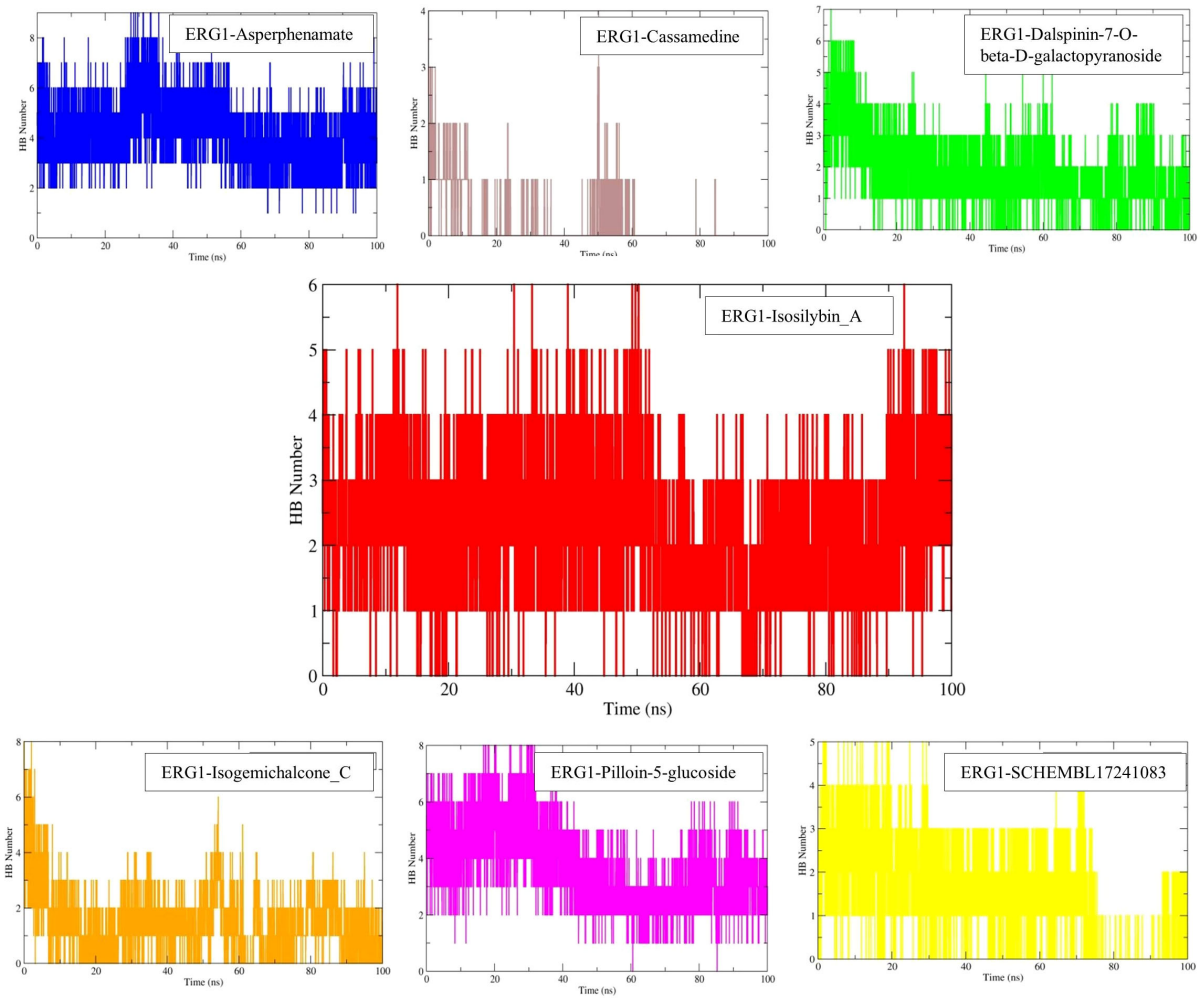
Ligand-protein hydrogen-bond counts over 100 ns. Mean ± SD values (20–100 ns): Asperphenamate-protein = 4.13 ± 1.27; Pilloin-5-glucoside-protein = 3.39 ± 1.30; Isosilybin A-protein = 2.31 ± 0.95; Dalspinin-7-O-β-D-galactopyranoside-protein = 1.65 ± 0.85; Isogemichalcone C-protein = 1.33 ± 0.89; SCHEMBL17241083-protein = 1.28 ± 1.02; Cassamedine-protein = 0.10 ± 0.35. Asperphenamate-protein and Pilloin-5-glucoside-protein maintained ≥1 H-bond in nearly all frames; Cassamedine-protein showed negligible polar contacts; whereas the Isosilybin A-protein maintained the most stable H-bonds over 100 ns.

Intermolecular hydrogen bonding varied among ligands (**Figure 7**). Asperphenamate (4.13 ± 1.27 H-bonds) and Pilloin-5-glucoside (3.39 ± 1.30 H-bonds) formed the highest number of H-bonds, with ≥1 H-bond present in nearly all frames. Isosilybin A however ranked high among leading 7 molecules for making averaged 2.31 ± 0.95 most persistent H-bonds, with occasional peaks of 4–6 bonds (≈10% of frames). Dalspinin-7-O-β-D-galactopyranoside and Isogemichalcone C displayed moderate counts (~1.3–1.7 H-bonds), while SCHEMBL17241083 averaged ~1.3 H-bonds. Cassamedine, however, formed negligible H-bonding (0.10 ± 0.35), with >90% of frames lacking any hydrogen bonds. The persistence of protein–ligand hydrogen bonding suggests stable ERG1 engagement, which may contribute to the observed adjuvant effect in subsequent *in vitro* assays.

#### 3.3.4 Conformational sampling (PCA)

**Figure 8:**
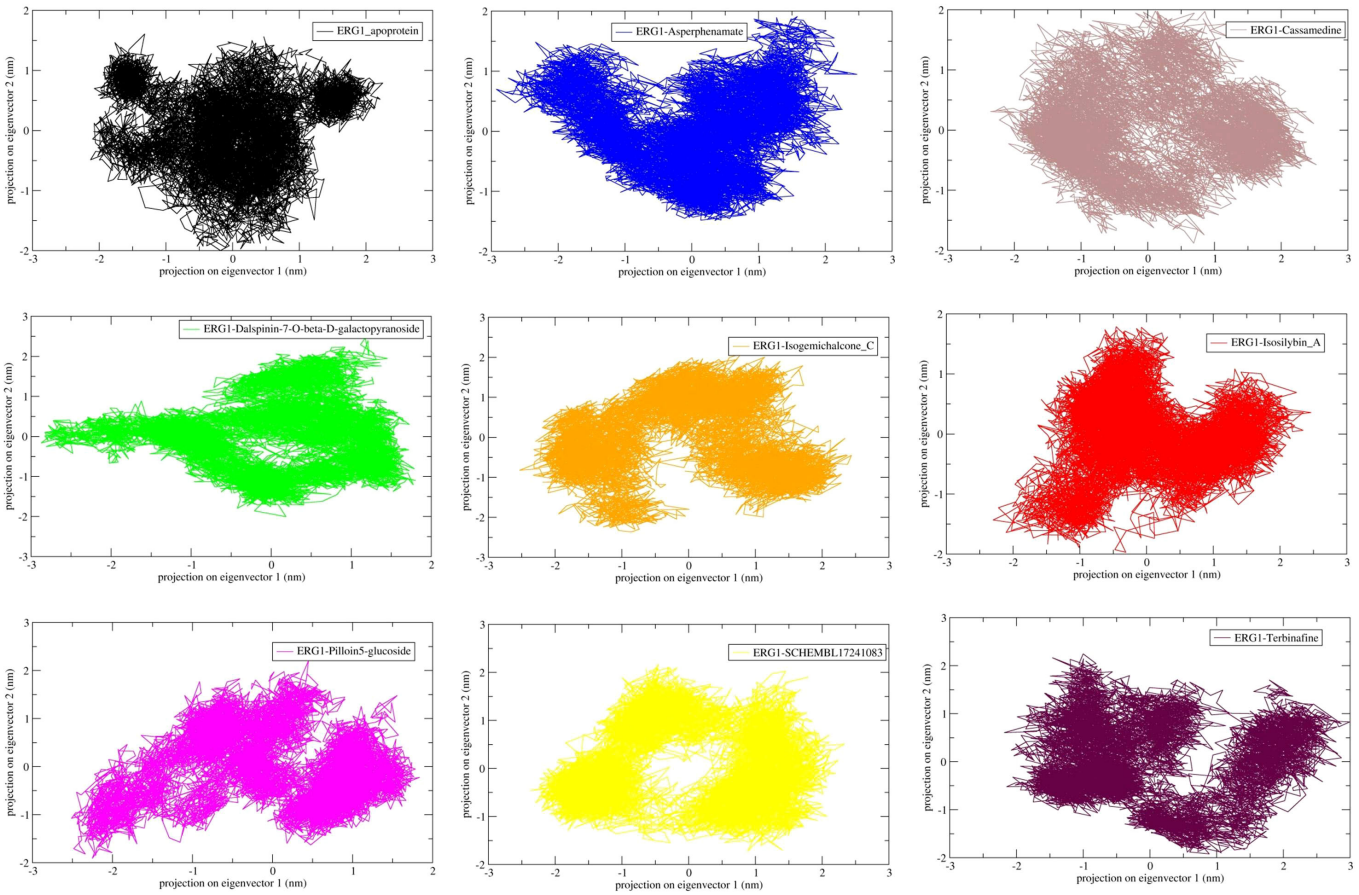
Principal component analysis (PCA) of backbone atomic fluctuations for apo ERG1 and ligand-bound complexes (100 ns). PC1–PC2 projections illustrate conformational sampling. Apo ERG1 explored a broad conformational space, while Isosilybin A, Pilloin-5-glucoside and Asperphenamate bound protein complexes clustered more compactly, consistent with stabilized dynamics. Cassamedine and Isogemichalcone C complexes exhibited broader dispersion, indicative of greater conformational heterogeneity.

Principal component analysis (PCA) of backbone atomic fluctuations was performed to evaluate conformational sampling along the dominant modes of motion. The apo ERG1 explored a broad conformational space along PC1–PC2, reflecting higher intrinsic flexibility. In contrast, Isosilybin A, Pilloin-5-glucoside and Asperphenamate displayed compact clustering in PC1–PC2 projections, suggesting that ligand binding restricts conformational motions and stabilizes ERG1 dynamics. Terbinafine exhibited moderate dispersion, comparable to the mid-range behaviour observed in RMSD and Rg analyses. Cassamedine and Isogemichalcone C showed more scattered distributions, indicating greater conformational heterogeneity and reduced stabilization of the protein fold (**Figure 8**). These PCA results are consistent with the global stability metrics (RMSD, Rg, SASA), flexibility profiles (RMSF), and H-bond interaction reinforcing the conclusion that the top candidate ligands constrain ERG1 dynamics more effectively than weaker binders.

Taken together, MD signatures (stable RMSD/Rg/SASA, reduced local RMSF near the pocket, persistent H-bonding and compact PCA clustering) identify Isosilybin A as the most promising phytochemical among those tested, with Pilloin-5-glucoside and Asperphenamate also showing favourable properties comparable to or in some respects better than terbinafine. Accordingly, further study was aimed to extract the Isosilybin A from its source plant *S. marianum* to validate the *in vitro* antifungal activity against test organisms.

#### 3.3.5 Binding free energy calculation (MM/PBSA)

The MM/PBSA method was used to further estimate the binding free energy of the ERG1-Isosilybin A complex. The calculated total binding free energy (ΔGbind) was −33.51 ± 3.98 kcal/mol, indicating that the ligand binds well to the ERG1 active site. This interaction was mainly driven by van der Waals forces (−55.11 kcal/mol) and electrostatic interactions (−26.17 kcal/mol), while polar solvation had a small opposing effect on binding (**Table 5**).

**Table 5:** Binding free energy decomposition of the ERG1-Isosilybin A complex calculated using the MM/PBSA method over 201 frames extracted from the 100 ns molecular dynamics trajectory.

Energy component	Average (kcal/mol)	SD
Van der Waals (ΔVDWAALS)	−55.11	3.06
Electrostatic (ΔEEL)	−26.17	5.24
Polar Solvation (ΔEGB)	+54.66	4.17
Non-polar Solvation (ΔESURF)	−6.89	0.38
Solvation Energy (ΔGSOLV)	+47.77	4.03
**Total Binding Free Energy (ΔGbind)**	**−33.51**	**3.98**

All energy values are reported in kcal/mol.

### 3.4 *In vitro* validation of the antifungal activity

#### 3.4.1 Qualitative antifungal assay

**Figure 9:**
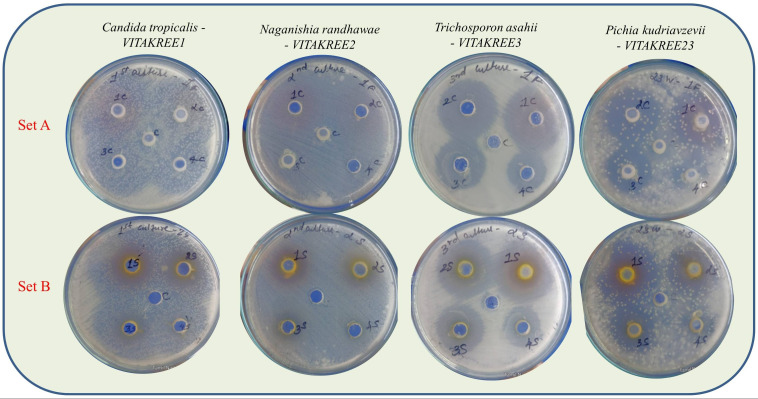
Agar well diffusion assay showing the antifungal activity of fluconazole alone (Set **(A)**) and fluconazole combined with *S. marianum* extract enriched with Isosilybin A (Set **(B)**) against selected yeast isolates. Fluconazole alone did not produce measurable zones of inhibition, indicating reduced susceptibility of the tested isolates. In contrast, the combination treatment produced clear, concentration-dependent inhibitory zones, suggesting enhanced antifungal responsiveness when fluconazole was administered alongside the Isosilybin A enriched extract.

The *in vitro* antifungal potential of fluconazole alone and its combination with *S. marianum* extract enriched with Isosilybin A was initially evaluated using an agar well diffusion assay (**Figure 9**). Fluconazole monotherapy (Set A) failed to produce measurable zones of inhibition against *C. tropicalis* (VITAKREE1), *Naganishia randhawae* (VITAKREE2), *Trichosporon asahii* (VITAKREE3), and *Pichia kudriavzevii* (VITAKREE23), with dense growth observed around the wells, indicating marked tolerance to fluconazole. In contrast, the combination treatment (Set B) produced clear, concentration-dependent inhibitory zones ranging from 20 to 25 mm across all tested isolates, suggesting an enhanced antifungal response when fluconazole was administered alongside the Isosilybin A enrich extract.

#### 3.4.2 Quantitative antifungal assay

To further substantiate these observations, a standardized microbroth dilution MIC assay was performed against nine yeast isolates, including *C. albicans* ATCC 10231 as the reference strain and environmental isolates comprising *C. tropicalis* (VITAKREE1), *Naganishia randhawae* (VITAKREE2), *Trichosporon asahii* (VITAKREE3), *Diutina rugosa* (VITAKREE7), *C. albicans* (VITAKREE8), *C. tropicalis* (VITAKREE19), and *Pichia kudriavzevii* (VITAKREE20 and VITAKREE23) (**Table 1**). The assay plates were arranged with a growth control well at the top, followed by seven wells in ascending order of concentration. The combination working solution was prepared by mixing fluconazole and the Isosilybin A enriched extract in a 1:1 ratio, followed by an additional 1:1 dilution with double-strength MHB during plate setup. Accordingly, the final effective concentration ranges were 0.19-12.5 μg/mL for fluconazole alone and 0.19-12.5 mg/mL for the extract in the combination treatment.

As illustrated in (**Supplementary Figure 1** in **Supplementary File 1**), fluconazole alone exhibited no inhibitory effect across the tested concentration range, as evidenced by persistent pink coloration following resazurin incubation, indicating sustained metabolic activity in all isolates. In contrast, the combination treatment resulted in a progressive reduction in metabolic activity, with complete growth inhibition consistently observed from the third dilution well onward. This corresponded to a concentration of 0.78 mg/mL of Isosilybin A enriched extract combined with 0.78 µg/mL of fluconazole, at which all isolates displayed stable purple/blue coloration, signifying metabolic arrest. Based on these observations, the MIC for the combination treatment was defined as the third dilution well for all tested isolates. While fluconazole alone was ineffective, its antifungal activity was markedly enhanced in the presence of the Isosilybin A enriched *S. marianum* extract.

#### 3.4.3 Fractional inhibitory concentration index

Because neither fluconazole nor the plant extract showed measurable MIC values when tested individually within the concentration range used, the FICI values could not be calculated using exact MIC values (Ng’uni et al., 2022). Therefore, the highest tested concentrations were used as surrogate MIC values for estimation. Based on this approximation, the calculated FICI values using standard formula suggested a strong synergistic interaction between fluconazole and the *S. marianum* extract (**Table 6**).

**Table 6:** FICI analysis of fluconazole in combination with *S. marianum* extract against resistant yeasts.

Isolate	MIC fluconazole alone (µg/mL)	MIC extract alone (mg/mL)	MIC fluconazole in combination (µg/mL)	MIC extract in combination (mg/mL)	FICI	Interpretation
Yeast isolates	>25	>25	0.78	0.78	0.0624	Synergy


$$FICI=\frac{MIC\;of\;drug\;alone}{MIC\;of\;drug\;in\;combo}+\frac{MIC\;of\;extract\;alone}{MIC\;of\;extract\;in\;combo\;}$$

## 4 Discussion

The increasing prevalence of antifungal resistance in *Candida* species and non-*Candida* environmental yeast isolates is a significant obstacle to successful treatment (Farmakiotis and Kontoyiannis, 2017; Whaley et al., 2017; Espinel-Ingroff et al., 2021). In this perspective, natural phytochemicals provide a compelling supply of structurally varied and physiologically active frameworks that might influence fungal physiology and improve antifungal medication efficacy (Li S. et al., 2018; Wink, 2022; Latif and Nawaz, 2025). This work illustrates that Isosilybin A, a principal component of *S. marianum*, serves as a powerful adjuvant that reinstates fluconazole sensitivity in resistant fungal strains. Through the integration of virtual screening, ADMET profiling, MD simulations, and *in vitro* synergy experiments, we present compelling evidence that natural metabolites may be utilized to combat antifungal resistance pathways.

A virtual screening of 17,967 phytochemicals against the ERG1 model showed seven lead compounds Asperphenamate, Cassamedine, Dalspinin-7-O-β-D-galactopyranoside, Isogemichalcone C, Pilloin-5-glucoside, SCHEMBL17241083, and Isosilybin A with docking scores above those of terbinafine. The phytochemicals established considerable van der Waals, hydrophobic, and polar contacts with critical ERG1 residues (ARG36, ARG43, VAL164, ASP200, ASP332), indicating significant pocket complementarity. Although docking alone is inadequate for prioritizing, the following ADMET filtering identified compounds with advantageous drug-likeness, solubility, and bioavailability. Cassamedine distinguished itself by meeting all five principal medicinal chemistry criteria (Lipinski, Ghose, Veber, Pfizer, GSK), whereas Isosilybin A provided an advantageous equilibrium of satisfactory bioavailability and stable anticipated interactions. This ADMET-guided selection highlights the significance of including pharmacokinetic characteristics early in natural product drug development processes.

MD simulations enhanced candidate prioritizing by assessing long-timescale structural stability and interaction durability. Isosilybin A, Asperphenamate, and Pilloin-5-glucoside demonstrated robust and constant hydrogen bonding, reduced local RMSF fluctuations at the active site, and compact PCA clustering patterns suggestive of pocket stabilization. The dynamic features indicate that many selected phytochemicals, including Isosilybin A, may have a mechanistic role in modifying ERG1 function or enhancing fungal cell sensitivity to antifungal drugs. Compounds like Cassamedine and Isogemichalcone C demonstrated stable global protein dynamics, indicating their potential as candidates for further biological testing.

A critical strength of this study is its testing against nine fungal isolates representing both clinical standards and environmental yeast pathogens: *C. albicans* ATCC 10231, *C. tropicalis* VITAKREE1, *Naganishia randhawae* VITAKREE2, *Trichosporon asahii* VITAKREE3, *Diutina rugosa* (syn. *C. rugosa*) VITAKREE7, *C. albicans* VITAKREE8, *C. tropicalis* VITAKREE19, and *Pichia kudriavzevii* VITAKREE20 and VITAKREE23. All isolates displayed multidrug resistance including complete non-responsiveness to fluconazole, amphotericin B, caspofungin, and flucytosine highlighting the real-world clinical difficulty posed by such organisms. The uniform restoration of fluconazole susceptibility across this diverse and highly resistant panel strengthens the biological significance of the observed synergy and confirms that the adjuvant effect of Isosilybin A is not isolate-specific but broadly effective across phylogenetically distinct yeast pathogens.

The *in vitro* investigations demonstrated the synergistic potential of Isosilybin A; while the extract’s independent antifungal efficacy was not validated. Fluconazole alone shown total ineffectiveness against all nine resistant isolates; however, its combination with the Isosilybin A rich extract reliably reinstated fungal growth suppression at the identical MIC dilution across all tested species. This repeatable synergy clearly indicates that Isosilybin A functions as an antifungal adjuvant, enhancing azole susceptibility rather than operating independently. The result reflects the established terbinafine fluconazole interaction (Barchiesi et al., 1997; Khodavandi et al., 2014); reinforcing the concept that Isosilybin A may affect ergosterol production or membrane-related susceptibility pathways. Future studies involving purified Isosilybin A and quantitative LC-MS analysis will be required to determine the exact concentration of the compound in the extract and its contribution to the observed antifungal activity.

This combined effect aligns with earlier findings concerning interactions between fluconazole and terbinafine; specifically, terbinafine increases azole susceptibility by interfering with ergosterol biosynthesis via the inhibition of squalene epoxidase (ERG1) (Barchiesi et al., 1997; Khodavandi et al., 2014). The present study did not perform a direct experimental comparison with terbinafine. Comparable methodologies designed to augment antifungal effectiveness have also been documented through structural or formulation-based optimization strategies. For example, the creation of econazole salt cocrystals has demonstrated improvements in both the physicochemical characteristics and antifungal activity of the original drug (Wang et al., 2025). Similarly, researchers have investigated bioactive compounds from plants as antifungal agents to combat drug-resistant *Candida*. Recent investigations have revealed that thymoquinone possesses the capacity to impede virulence-related factors, including biofilm formation and metabolic enzymes, within *C. albicans* (Khan et al., 2024). This action subsequently diminishes fungal pathogenicity and resistance. Furthermore, liposomal formulations of thymoquinone have exhibited improved therapeutic effectiveness against fluconazole-resistant *C. albicans in vivo* (Khan et al., 2015). In alignment with these findings, the augmented antifungal response observed in the current study implies that Isosilybin A may function as a phytochemical adjuvant, thereby enhancing fluconazole responsiveness, rather than serving as a direct substitute for terbinafine. These results, rather than indicating robust intrinsic antifungal activity, suggest that Isosilybin A may facilitate the re-sensitization of fluconazole-resistant yeast isolates. While computational assessments indicate a potential interaction between Isosilybin A and the ERG1 active site, this study did not include direct experimental confirmation of ERG1 inhibition. To definitively establish the precise molecular mechanism of action, assays like ergosterol quantification or ERG1 gene expression analysis would be necessary. The suggested interaction between Isosilybin A and ERG1 should be regarded as a computational prediction, warranting additional experimental validation. Consequently, the current *in vitro* data collectively support the potential application of the Isosilybin A rich *S. marianum* extract as an adjunctive strategy to augment fluconazole efficacy against resistant *Candida* and emerging non-*Candida* yeast species.

Importantly, the remaining six phytochemicals shortlisted through *in silico* screening and ADMET evaluation represent promising future leads. Their strong predicted interactions, favorable stability in MD simulations and in several cases superior ADMET properties warrant further *in vitro* and *in vivo* exploration. Validating these phytochemicals experimentally may uncover additional adjuvant molecules or even direct antifungal agents capable of targeting ERG1 or other resistance-related pathways. Such systematic validation would not only expand the repertoire of natural antifungal candidates but also help identify combinatorial therapies that can be leveraged against resistant fungal pathogens.

The amalgamation of computational screening, pharmacokinetic assessment, dynamic modeling, and synergy-focused biological testing underscores the significance of phytochemicals as compounds that enhance therapeutic efficacy. Isosilybin A now serves as a prominent adjuvant, while the wider array of natural metabolites revealed in this work provides a potential basis for next-generation antifungal tactics to combat drug resistance and enhance therapeutic efficacy.

## 5 Conclusion

This research combines computational prediction with experimental confirmation to show that natural phytochemicals specifically Isosilybin A from *S. marianum* can augment the antifungal effectiveness of standard medications and assist in overcoming resistance in clinically significant yeasts. The extract’s independent antifungal efficacy was not demonstrated; nevertheless, its strong synergistic effect with fluconazole reliably reinstated susceptibility in all nine resistant isolates, underscoring its role as a natural adjuvant rather than a standalone therapeutic agent. Isosilybin A, via interacting with ERG1, which has high anticipated stability *in silico* studies and facilitates concentration-dependent suppression in combination therapy *in vitro*, presents itself as a prospective regulator of ergosterol production, potentially enhancing current antifungal treatments. These findings highlight the significance of plant-derived metabolites as effective adjunct compounds that can resensitize resistant fungal infections, providing a promising avenue for the development of next-generation combination antifungal therapies.

## Data Availability

The datasets presented in this study can be found in online repositories. The names of the repository/repositories and accession number(s) can be found below: https://www.ncbi.nlm.nih.gov/, VITAKREE1, VITAKREE2, VITAKREE3, VITAKREE7, VITAKREE8, VITAKREE19, VITAKREE20, VITAKREE23.
