# Supplementary material for: In silico identification of isosilybin a targeting squalene epoxidase as an antifungal adjuvant with in vitro validation against azole-resistant Candida and emerging yeasts
Source: Front Cell Infect Microbiol. 2026 May 12;16:1774455. doi: 10.3389/fcimb.2026.1774455 (PMC13201520; doi:10.3389/fcimb.2026.1774455)
Supplement: Supplementary file 1 [file DataSheet1.pdf]

Supplementary file for article
"*In silico* Identification of Isosilybin A Targeting Squalene Epoxidase (ERG1) as an Antifungal Adjuvant with *in vitro* Validation Against Azole-Resistant *Candida* and Emerging Yeasts"

Table 1: Configuration used for molecular docking for ERG1 protein and phytochemical ligands

	center_x	center_y	center_z	size_x	size_y	size_z	energy_range	exhaustiveness	num_modes
For total phytochemicals	–5.278	–0.431	–1.159	30	30	30	3	8	9
For top 500 phytochemicals	–5.278	–0.431	–1.159	30	30	30	3	50	20

Table 4: Residue-level interaction profile of selected phytochemicals and terbinafine with the ERG1 active site based on molecular docking analysis.

Ligand	Van der Waals Interactions	Hydrophobic Interactions	Polar Interactions	Hydrogen Bond Interactions
**Asperphenamate**	GLY12, GLY14, GLU35, HIS140, CYS199, GLY201, GLY331, PRO339, GLY344, MET345, GLY348	VAL15, ILE16, VAL45, ILE202, TYR203	ARG36, ARG43, MET309, ASP332	Conventional H-bonds: ARG36, ARG43, ASP200, ASP332
**Cassamedine**	ILE11, ALA13, GLY14, VAL34, GLU35, HIS140, ALA162, THR163, CYS199, GLY201, ILE202, TYR203, LYS205, PHE206, MET345, GLY348	VAL15, ILE16, ASP200	GLY12, ARG36, ARG43, VAL164, GLY331, ASP332, GLY344	Conventional H-bonds: VAL164, ASP332; Carbon H-bonds: GLY12, ARG36, GLY331, GLY344
**Dalspinin-7-O-β-D-**	ILE11, GLY12, ALA13, GLY14, VAL45, GLY46,	ARG43	VAL15, GLU35,	Conventional H-bonds: VAL15,

**galactopyranoside**	HIS140, THR163, VAL164, GLY201, ILE202, TYR203, LYS205, ASP332, MET345		ARG36, ARG43, ASP200	GLU35, ARG43; Carbon H-bonds: ARG36, ASP200
**Isogemichalcone C**	ILE11, GLY14, VAL15, ILE16, VAL34, GLU35, ASP37, HIS140, THR163, CYS199, ASP200, TYR203, LYS205, PHE206, MET309, GLN312, ASP332, ARG337, PRO339	ARG36, VAL45, GLY201, ILE202, MET336	GLY12, ARG36, ARG43, VAL164, PRO310, ASN311	Conventional H-bonds: ARG36, ARG43, VAL164, PRO310; Carbon H-bonds: GLY12, ASN311
**Isosilybin A**	ILE11, ALA13, GLY14, ILE16, VAL34, ARG36, VAL45, THR163, CYS199, GLY201, ILE202, SER204, LYS205, PHE206, GLY344, ASP332	VAL15, ARG43, MET345	GLY12, GLU35, ARG43, VAL164, ASP200, TYR203	Conventional H-bonds: GLU35, ARG43, VAL164, TYR203; Carbon H-bonds: GLY12, ASP200
**SCHEMBL17241083**	ILE11, GLY12, GLY14, ILE16, VAL34, GLY46, GLU35, HIS140, ALA162, THR163, VAL164, CYS199, GLY201, ILE202, TYR203, LYS205, PHE206, MET309, GLY344	VAL15, ARG36, VAL45, MET345	ARG36, ARG43, ASP200, ASP332	Conventional H-bonds: ARG36, ARG43; Carbon H-bonds: ASP200
**Pilloin-5-glucoside**	ILE11, GLY14, VAL34, ALA162, THR163, VAL164, ASP200, GLY201, PHE206	ARG36, ILE202, TYR203	GLY12, GLU35, ARG36, ARG43, LYS205	Conventional H-bonds: GLY12, ARG43; Carbon H-bonds: LYS205
**Terbinafine**	ILE11, GLY12, GLY14, VAL15, VAL34, GLU35, ARG43, HIS140, ALA162, THR163, VAL164, CYS199, GLY201, ILE202, TYR203, LYS205, ASP332	ARG36	ASP200	Carbon H-bond: ASP200

Table 5: Drug likeness and ADMET properties of leading 7 compounds

**Phytochemical name**	Asperphenamate	Dalspinin-7-O-beta-D-galactopyranoside	SCHEMBL17241083	Isogemichalcone C	Pilloin 5-glucoside	Isosilybin A	Cassamedine
Lipinski filter	Passed	Passed	Passed	Passed	Passed	Passed	Passed
Ghose filter	Failed	Failed	Passed	Failed	Passed	Failed	Passed
Veber filter	Bad	Bad	Good	Bad	Bad	Bad	Good
Pfizer filter	Bad	Good	Bad	Bad	Good	Good	Good
GSK filter	Bad	Bad	Bad	Bad	Bad	Bad	Good
QED score	0.2911	0.3259	0.651	0.1065	0.3331	0.3742	0.5228
Bioavailability score	0.17	0.55	0.55	0.55	0.55	0.55	0.55
Solubility class [ESOL]	Poorly soluble	Soluble	Moderately soluble	Poorly soluble	Soluble	Moderately soluble	Moderately soluble
Solubility class [Silicos-IT]	Insoluble	Soluble	Moderately soluble	Moderately soluble	Soluble	Moderately soluble	Moderately soluble
BBB permeation	No	No	No	NO	No	No	Yes
GI absorption	High	Low	High	Low	Low	Low	High

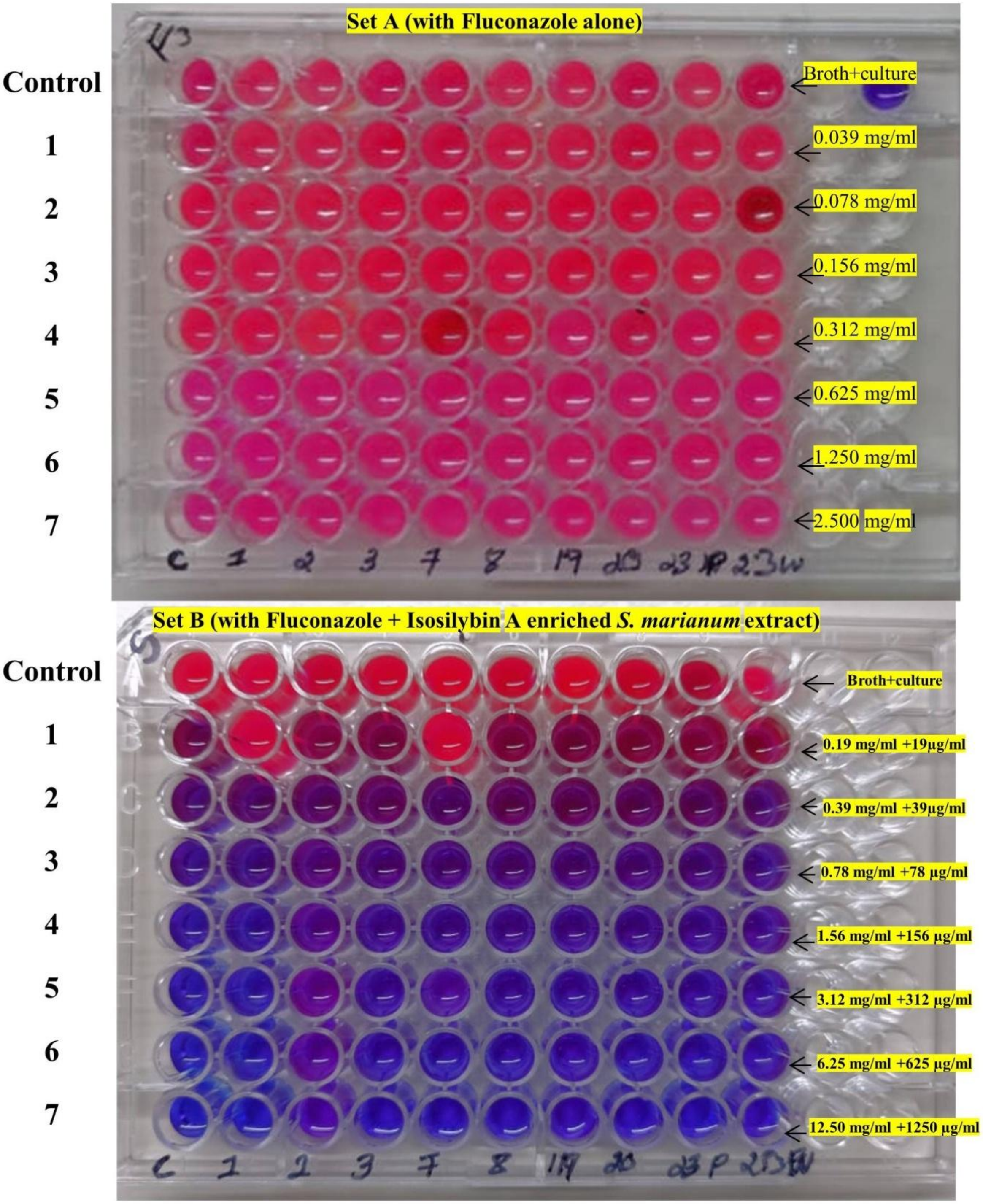

Figure 10: Micro broth dilution MIC assay of fluconazole alone (Set A) and its combination with Isosilybin A enriched *S. marianum* extract (Set B) against nine yeast isolates.

Note: Wells were arranged in ascending order of concentration from top (growth control) to bottom (highest concentration). Fluconazole alone showed no growth inhibition across the tested concentration range, as indicated by persistent pink coloration following resazurin incubation. The combination treatment demonstrated complete metabolic inhibition beginning at the third dilution well, reflected by stable purple/blue coloration, indicating enhanced fluconazole activity in the presence of the extract.
